# Effects of home-based long-term care services on caregiver health according to age

**DOI:** 10.1186/s12955-017-0786-6

**Published:** 2017-10-23

**Authors:** Ming-Chun Chen, Chi-Wen Kao, Yu-Lung Chiu, Tzu-Ying Lin, Yu-Ting Tsai, Yi-Ting Zhang Jian, Ya-Mei Tzeng, Fu-Gong Lin, Shu-Ling Hwang, Shan-Ru Li, Senyeong Kao

**Affiliations:** 1 Graduate Institute of Life Science, National Defense Medical Center, Taipei, Taiwan, Republic of China; 2 Department of Superintendent’s Office, Yang Ji Hospital, Keelung, Taiwan, Republic of China; 3 Nursing Department, Tri-Service General Hospital; School of Nursing, National Defense Medical Center, Taipei, Taiwan, Republic of China; 4 School of Public Health, National Defense Medical Center, No. 161, Min-Chun E. Rd., Sec. 6, Taipei, Taiwan, Republic of China; 5 Department of Optometry, University of Kang Ning, Taipei, Taiwan, Republic of China; 6 Center for General Education, National Defense Medical Center, Taipei, Taiwan, Republic of China

**Keywords:** Caregiver, Long-term care (LTC), Self-rated health (SRH), Home-based care

## Abstract

**Background:**

Caregiver health is a crucial public health concern due to the increasing number of elderly people with disabilities. Elderly caregivers are more likely to have poorer health and be a care recipient than younger caregivers. The Taiwan government offers home-based long-term care (LTC) services to provide formal care and decrease the burden of caregivers. This study examined the effects of home-based LTC services on caregiver health according to caregiver age.

**Methods:**

This cross-sectional study included a simple random sample of care recipients and their caregivers. The care recipients had used LTC services under the Ten-Year Long-Term Care Project (TLTCP) in Taiwan. Data were collected through self-administered questionnaires from September 2012 to January 2013. The following variables were assessed for caregivers: health, sex, marital status, education level, relationship with care recipient, quality of relationship with care recipient, job, household monthly income, family income spent on caring for the care recipient (%) and caregiving period. Furthermore, the following factors were assessed for care recipients: age, sex, marital status, education level, living alone, number of family members living with the care recipient, quality of relationship with family and dependency level. The health of the caregivers and care recipients was measured using a self-rated question (self-rated health [SRH] was rated as very poor, poor, fair, good and very good).

**Results:**

The study revealed that home nursing care was significantly associated with the health of caregivers aged 65 years or older; however, caregivers aged less than 65 who had used home nursing care, rehabilitation or respite care had poorer health than those who had not used these services. In addition, the following variables significantly improved the health of caregivers aged 65 years or older: caregiver employment, 20% or less of family income spent on caregiving than 81%–100% and higher care recipient health. The involvement of daughters-in-law, rather than spouses, and care recipient health were positively related to the health of caregivers aged less than 65 years.

**Conclusions:**

The findings suggest that home-based LTC service use benefits the health of elderly caregivers. By contrast, home-based LTC service use may be negatively correlated with the health of the caregivers aged less than 65 years.

**Electronic supplementary material:**

The online version of this article (10.1186/s12955-017-0786-6) contains supplementary material, which is available to authorized users.

## Background

The worldwide rate of people aged 65 years or older in developing countries is expected to increase to approximately 20% by 2050 [1]. An increasing number of family members have become informal caregivers and experience a considerable caring burden and stress, because they care for elderly people with disabilities [2–4]. Many studies have reported informal caregivers have a higher risk of illness and mortality than noncaregivers [5–7]. Caregiver health has become a crucial public health concern in several countries, owing to the marked increase in informal caregivers [8]. Many countries assist caregivers and reduce their burden by establishing long-term care (LTC) systems that provide services at the societal level; this can even improve the caregiver’s health [9, 10]. Two meta-analyses by Pinquart and Sorensen [11, 12] indicated that formal care support did not affect caregiver health. In these two studies, formal care was defined as support from professional helpers other than relatives and neighbours, but the supports were not necessarily provided by governments at the societal level. In addition, caregiver health was assessed by subjective and objective methods. Subjective health could be evaluated by a single-item indicator and symptom checklists (e. g., Short Form 36 health survey, SF-36); objective health could be assessed by the number of medical conditions, hospitalizations or doctor visits and medication use. Additionally, in the United States, Japan and South Korea, studies associated with the effects of LTC services at the societal level have only focused on caregiver stress and burden, and the results were uncertain [13, 14]. Two studies in the United States and Japan have shown that LTC services significantly reduced caregiver stress and burden [13, 14]; however, a study in Japan found that formal LTC services were not associated with reduced stress among family caregivers [15]. Therefore, additional studies are required to evaluate the effects of LTC services on caregiver health.

Besides formal services, many factors were related to caregiver health. Pinquart and Sorensen [12] integrated the outcomes of 176 studies to present variables related to the health of informal caregivers who cared for older those receiving care. They suggested that older age, not being a spouse, coresidence, duration of caregiving, lower education, lower income and less informal support were associated with worse health. However, physical function of care recipients was not related to caregiver health. Schulz & Sherwood [15] reviewed studies exploring the health effects of family caregiving and concluded that sex, age, relationship with care recipients, duration of caring and care recipients’ functional disability were linked to caregiver health. Richardson, Lee, Berg-Weger, & Grossberg [6] reviewed relevant research studies on the health of caregivers of patients with Alzheimer’s disease and dementias. They concluded that variables associated with health were care-recipient behavioural problems, duration of caregiving, age, relationship with the care recipient and socio-economic status. A survey reported that working was a protector and inadequate income was a risk factor for caregiver health [10]. In conclusion, caregiver health was related to the above-mentioned caregivers’ and care recipients’ factors.

As the population ages, more care recipients are aged 85 years or older and their caregivers are aged 65 years or older [16]. Caregiver age is a crucial characteristic for the following reasons. First, elderly caregivers are potential care recipients because they report poorer health compared with younger caregivers [12, 17, 18]. Second, people older than 65 are the main users of LTC services [19]. Furthermore, these elderly care recipients may care for each other because of changes in family structure and the traditional value of filial piety [20–22]. Third, most people retire after turning 65 and may have no family or work conflict preventing them from becoming caregivers.

Taiwan is one of the most rapidly aging countries, and the number of people with disabilities is expected to increase twofold in the next 20 years [23, 24]. Accordingly, many family members must provide care for them. Therefore, in 2007, the Taiwan government devised the Ten-Year Long-Term Care Project (TLTCP) at the societal level to provide appropriate services to people with disabilities; thereby mitigating the burden of family caregivers. LTC was defined as caring for people with disabilities and categorized into three dependence levels evaluated by the items of difficulty in activities of daily living (ADLs) and instrumental activities of daily living (IADLs). In addition, four home-based LTC services are provided by the TLTCP: home services, rehabilitation, nursing care and respite care [25]. However, no information is available regarding the effects of home-based LTC services on caregiver health in Taiwan. Therefore, this study examined whether the use of home-based LTC services is associated with the health of elderly and younger caregivers, and compared the factors related to caregivers’ health between both age groups.

## Methods

### Design

This cross-sectional study examined the effects of home-based LTC service use on caregiver health. The research protocol was approved by the Institutional Review Board of the Tri-Service General Hospital, National Defense Medical Center, Taipei, Taiwan (1–101–05-038).

### Sample and data collection

We enrolled people who registered and used the LTC services of the TLTCP and their caregivers between January 2011 and April 2012 in Taipei, Taiwan. The following people fulfilled the eligibility criteria for receiving the LTC services of the TLTCP and were recruited for this study: aboriginal people aged 55 years or older, people with a disability aged 50 years or older, and all people aged 65 years or older. A caregiver was defined as a person aged 18 years or older who mainly cared for an LTC service user and was not paid for doing so [25]. Through simple random sampling, we selected a sample of care recipients from 6831 LTC service users and their caregivers, from the LTC dataset of the TLTCP. G*power version 3.1.6 was used to determine the sample sizes for linear multiple regression analysis with α = 0.05, 80% power, 22 predictors, and effect size = 0.058 (14). The minimum sample size was 393 pairs of participants. Given an estimated response rate of 40%, the sample was estimated to be increased to approximately 983 pairs of caregivers and care recipients.

Before the current study, we conducted a pilot study to establish the reliability of the data collection instrument using a separate sample of 30 care recipients and caregivers, separately. The questionnaire items were not changed because the pilot test results revealed a high degree of response consistency (care recipients: 96%, caregivers: 99%) for these items. Then we contacted care recipients via telephone to explain the purpose of the study and the criteria of main caregivers. The caregivers were identified by care recipients. We mailed two versions of the self-administered questionnaires with different contents (one version each for the care recipients and caregivers; Additional file 1: Appendix A) and an informed consent form to each care recipient for data collection. The questionnaires were first provided to potential participants between September 2012 and January 2013 to evaluate the immediate effects of home-based LTC services on the caregivers’ health. After 2 weeks, people who did not return the questionnaires were reminded by telephone to complete the questionnaire. A complete questionnaire was defined as providing an answer for caregivers’ health and age. Overall, 598 pairs of caregivers and recipients returned completed questionnaires: the response rate was 60.8%. There were no statistical differences between responders and non-responders among care recipients regarding age and sex (data not shown).

## Measures

### Independent variables

In the TLTCP, care recipients were provided with 11 types of LTC services based on their dependence levels evaluated by ADLs and IADLs. Of the 11 LTC services, 4 were home-based LTC services. Home-based LTC services include home nursing care by a registered nurse (change catheters, draw blood, etc.); home rehabilitation by a physical therapist, rehabilitation physician, or occupational therapist (muscle strength, endurance training, etc.); home respite care by a home care attendant (temporary personal or nursing care) and home service (bathing, walking, dressing, toileting, etc.). The care recipients were asked the following questions, “Have you ever used home nursing care?”, “Have you ever used home rehabilitation?”, “Have you ever used home respite care?” and “Have you ever used home service?” The answer for each question was yes or no. The care recipients who answered “no” were considered the reference group for each “use of home-based services” question.

### Dependent variables

The dependent variable was the self-rated health (SRH) of the caregivers, which is a subjective indicator of overall health status-related physical and mental health [11]; it has been reported to predict mortality [26, 27]. The test–retest reliability of single-item SRH was good for ages 18 to 73 (kappa values of 0.64–0.73) [28]. Caregiver health was evaluated using the question, “How would you rate your health generally since the care recipient started receiving LTC services?” The answer options were very poor, poor, fair, good and very good. SRH was treated as a continuous variable and considered to be close to equally spaced in this study, according to the previous studies [29]. Additionally, a prior study indicated that SRH formed a continuum from poor to good health, showing a similar pattern to the categorical form: average and poor/very poor compared with good/excellent self-rated health [30].

### Covariates

Covariates were characteristics of the care recipients and caregivers that were related to caregiver health. They were based on those of previous studies [6, 10, 12, 31]. The analysed caregiver characteristics were as follows: sex (male or female), marital status (unmarried or married), education level (less than or equal to elementary school, middle school, high school, or college or above), relationship with care recipient (spouse, son, daughter, daughter-in-law or other), quality of relationship with care recipient (very bad, bad, good or very good), job (yes or no), household monthly income (NTD; <30,000, 30,000–69,999, or ≥70,000), family income spent on caring for the care recipient (%; ≤20%, 21%–40%, 41%–60%, 61%–80%, or 81%–100%) and caregiving period (in years). The following characteristics of the care recipients were analysed: age (<65, 65–74, 75–84, or ≥85 years), sex (male or female), marital status (unmarried or married), education level (illiterate, literate/primary school, or junior high or above), living alone (yes or no), number of family members living with the care recipient (0, 1–3, or ≥4), quality of relationship with family (very bad, bad, good, or very good), dependency level (intact, mild, moderate, or severe), and SRH (1–5, where 1 = very poor and 5 = very good). The dependency level of the care recipients was evaluated using ADLs and instrumental IADLs. Care recipients with an intact level did not experience difficulty in any ADL or IADL; a low level is defined as care recipients who live alone and experience difficulty in 1 or 2 ADL items or difficulty in any IADL item. The moderate and high dependency levels are identified when care recipients experience difficulty in 3 or 4 and 5 or 6 ADL items, respectively [25].

## Statistical analyses

Data were analysed using SPSS 22.0. Continuous variables were represented as means and standard deviation; categorical variables were represented as frequencies and percentages. The chi-squared and independent t tests were used for comparing the characteristics of caregivers aged 65 years or older and aged less than 65 years. We used univariate linear regression to select the potential covariates and multivariable linear regression to evaluate the effects of home-based LTC service use on caregiver health after adjustment for all the possible covariates. Before the analysis, regression diagnostics were performed: linearity, no multicollinearity, and homogeneity as well as normality and independence of residuals. The linear relationship between SRH and the independent variables was tested using the one-way analysis of variance test of linearity. A scatter plot of the standardized residuals versus the predicted Y′ values was generated to check the homoscedasticity of variance. Normality was checked using the Kolmogorov–Smirnov goodness test. The independence of residuals was checked by the Durbin–Watson test. Multicollinearity was checked by the variance inflation factor (VIF), with VIF > 10 indicating the possibility of multicollinearity among the independent variables. The data in our study met the assumptions of multivariable linear regression. Statistical significance was set at *p* < 0.05 for all the tests.

## Results

### Characteristics of caregivers and care recipients

Table 1 summarizes participant characteristics. Many differences were observed between the caregivers aged less than aged 65 years and those aged 65 years or older. Among all the 598 participant pairs, the caregivers aged 65 years or older were, compared with the caregivers aged less than 65 years, more likely to have a lower SRH after LTC service use (2.67 vs. 2.86, *p* = 0.047), be married (96.4% vs. 80.7%, *p* < 0.001), have a middle school education or less (34.8% vs. 10.6%, *p* < 0.001), be a spouse (71.0% vs. 11.1%, *p* < 0.001), be unemployed (92.0% vs. 48.2%, *p* < 0.001), have an income lower than NTD70,000 (71.0% vs. 57.3%, *p* = 0.013), and spend more than 40% of family income for care (52.4% vs. 39.4%, *p* = 0.029). The characteristics of care recipients cared by caregivers less than aged 65 and those cared by caregivers aged 65 years or older differed significantly in sex, education level, number of family members living with them and physical function. Care recipients receiving care from caregivers aged 65 years or older were more likely to be men (58.3% vs. 42.0%, *p* = 0.002), have a junior high education or above (60.8% vs. 44.2%, *p* = 0.002), have 1 to 3 family members living with them (69.2% vs. 45.1%, *p* < 0.001), and have an intact or mild dependency level (36.7% vs. 20.1%, *p* = 0.002), compared with those receiving care from caregivers less than 65 years.

**Table 1:** Characteristics of participants stratified by caregiver age

Variables	Caregivers aged less than 65 *n* = 460	Caregivers aged 65 or older *n* = 138	*p*-value
	n (%) / Mean ± SD	n (%) / Mean ± SD	
Family caregivers			
SRH after service use ^a^	2.86 ± 0.95	2.67 ± 0.98	0.047
Sex			0.709
Male	141 (30.7)	40 (29.0)	
Female	319 (69.3)	98 (71.0)	
Marital status			<0.001
Unmarried	88 (19.3)	5 (3.6)	
Married	369 (80.7)	132 (96.4)	
Education level			<0.001
≤ Elementary school	20 (4.3)	21 (15.2)	
Middle school	29 (6.3)	27 (19.6)	
High school	144 (31.3)	44 (31.9)	
≥ College	267 (58.0)	46 (33.3)	
Relationship with care recipient			<0.001
Spouse	51 (11.1)	98 (71.0)	
Son	133 (29.0)	17 (12.3)	
Daughter	179 (39.0)	14 (10.1)	
Daughter-in-law	71 (15.5)	2 (1.4)	
Others ^b^	25 (5.4)	7 (5.1)	
Quality of relationship with care recipient			0.982
Very bad	5 (1.1)	1 (0.7)	
Bad	12 (2.6)	4 (2.9)	
Good	181 (39.9)	54 (39.7)	
Very good	256 (56.4)	77 (56.6)	
Job			<0.001
No	220 (48.2)	127 (92.0)	
Yes	236 (51.8)	11 (8.0)	
Household monthly income (NTD) ^c^			0.013
< 30,000	53 (15.1)	21 (27.6)	
30,000–69,999	148 (42.2)	33 (43.4)	
≥ 70,000	150 (42.7)	22 (28.9)	
Family income spent on caring for the care recipient (%)			0.029
20 or below	126 (30.1)	25 (20.2)	
21–40	128 (30.5)	34 (27.4)	
41–60	103 (24.6)	34 (27.4)	
61–80	34 (8.1)	14 (11.3)	
81–100	28 (6.7)	17 (13.7)	
Caregiving period (years)	6.30 ± 5.67	7.41 ± 6.45	0.060
Care recipients			
Age			0.466
65 or less	39 (9.5)	6 (5.0)	
65–74	71 (17.3)	23 (19.3)	
75–84	151 (36.8)	47 (39.5)	
85 or older	149 (36.3)	43 (36.1)	
Sex			0.002
Male	174 (42.0)	70 (58.3)	
Female	240 (58.0)	50 (41.7)	
Marital status			0.583
Unmarried	14 (3.4)	6 (5.0)	
Married	400 (96.6)	114 (95.0)	
Education level			0.002
Illiterate	100 (24.3)	14 (11.7)	
Literate/Primary school	130 (31.6)	33 (27.5)	
Junior high and above	182 (44.2)	73 (60.8)	
Living alone			0.051
No	355 (85.7)	111 (92.5)	
Yes	59 (14.3)	9 (7.5)	
Number of family members living with care recipient			<0.001
0	59 (14.5)	9 (7.5)	
1–3	184 (45.1)	83 (69.2)	
4 or more	165 (40.4)	28 (23.3)	
Quality of relationship with family			0.561
Very bad	2 (0.5)	2 (1.7)	
Bad	14 (3.4)	3 (2.5)	
Good	182 (44.7)	55 (46.2)	
Very good	209 (51.4)	59 (49.6)	
Dependency level			0.002
Intact	21 (5.1)	14 (11.7)	
Low	62 (15.0)	30 (25.0)	
Moderate	284 (68.6)	65 (54.2)	
High	47 (11.4)	11 (9.2)	
SRH	2.22 ± 0.95	2.32 ± 0.93	0.324
Use of home-based services			
Home nursing care	180 (43.5)	43 (35.8)	0.135
Home rehabilitation	98 (23.8)	23 (19.2)	0.299
Home respite care	55 (13.3)	14 (11.7)	0.642
Home service	192 (46.4)	74 (61.7)	0.003

*p*-value was examined using chi-squared and independent *t* tests. ^a^ was rated from 1 (very bad) to 5 (very good). ^b^ Others: son-in-law, grandchild, brother, sister, etc. ^c^ 30,000 NTD equals approximately 1000 USD. *SD* standard deviation, *SRH* self-rated health

### Home-based LTC service use

Table 1 shows information on home care service use among the caregivers by the chi-squared test. The care recipients cared by caregivers aged 65 years or older used more home service than did those cared by caregivers aged less than 65 years (61.7% vs. 46.4%, *p* = 0.003). However, the proportions of care utilization for home nursing care, rehabilitation and respite care did not differ significantly among the care recipients cared by caregivers of both age groups.

### Effects of home-based LTC care service use on caregiver health

We examined the factors determining the association between the independent factors and SRH among caregivers aged younger than 65 years by using univariate and multivariable linear regression (Additional file 2: Appendix B; Table 2). In the univariate linear regression model, the relationship with the care recipient; caregiver’s working status; the caregiver’s monthly household income; family income spent on caring for the care recipient (%); number of family members living with the care recipient; SRH of the care recipient; and use of home nursing care, rehabilitation and respite care were significantly correlated with the health of caregivers aged less than 65 years. Advance, variables that were significantly associated in the univariate linear regression were included in the multivariable linear regression. The results revealed that the relationship with the care recipient; SRH of the care recipient; and use of home nursing care, rehabilitation, and respite care were significantly correlated with caregiver health in the multivariable analysis. Caregivers aged less than 65 years who took responsibility for care recipients who had used home nursing care, rehabilitation and respite care had significantly lower SRH scores of 0.229, 0.265 and 0.413 points—adjusted for covariates—respectively, compared with those who took responsibility for care recipients who had not used each of these services. However, daughters-in-law showed higher SRH scores than spouses (β=0.538, 95% CI = 0.129–0.948) among caregivers aged less than 65 years, after adjustment for independent and covariates. In addition, caregivers aged less than 65 years exhibited a higher SRH score when their care recipients had a more favourable SRH score (β=0.127, 95% CI = 0.018–0.236), after adjustment for independent and covariates.

**Table 2:** Multivariable linear regression of effects of home-based long-term care service use on self-rated health among caregivers stratified by caregiver age

Variables	Caregivers aged less than 65 n = 460	Caregivers aged 65 or older n = 138
	β (95% CI)	β (95% CI)
Family caregivers		
Relationship with care recipient		
Spouse	Reference	–
Son	0.226 (−0.146, 0.597)	–
Daughter	0.209 (−0.155, 0.572)	–
Daughter-in-law	0.538 (0.129, 0.948)^*^	–
Others ^a^	0.203 (−0.366, 0.772)	–
Job		
Yes	Reference	Reference
No	0.203 (−0.22, 0.429)	0.671 (0.044, 1.299)^*^
Household monthly income (NTD) ^b^		
< 30,000	Reference	–
30,000–69,999	0.078 (−0.246, 0.402)	–
≥ 70,000	0.266 (−0.076, 0.609)	–
Family income spent on caring for the care recipient (%)		
20 or below	Reference	Reference
21–40	−0.172 (−0.439, 0.095)	0.031 (−0.455, 0.518)
41–60	−0.057 (−0.349, 0.236)	−0.455 (−0.939, 0.029)
61–80	−0.135 (−0.610, 0.341)	−0.060 (−0.671, 0.552)
81–100	−0.361 (−0.782, 0.060)	−0.948 (−1.534, −0.361)^**^
Caregiving period (years)		
Care recipients		
Number of family members living with care recipient		
0	Reference	–
1–3	0.016 (−0.320, 0.352)	–
4 or more	−0.044 (−0.384, 0.296)	–
SRH	0.127 (0.018, 0.236)^*^	0.222 (0.038, 0.406)^*^
Use of home-based services		
Home nursing care	−0.229 (−0.447, −0.010)^*^	0.499 (0.146, 0.852)^**^
Home rehabilitation	−0.265 (−0.513, −0.016)^*^	–
Home respite care	−0.413 (−0.722, −0.105)^**^	–
Home service	0.048 (−0.174, 0.270)	–

^a^ Others: son-in-law, grandchild, brother, sister, etc. ^b^ 30,000 NTD equals approximately 1000 USD. *CI* confidence interval, *SRH* self-rated health. *p < 0.05; **p < 0.01

The caregiver’s working status, family income spent on caring for the care recipient (%), care recipient SRH, and home nursing care use were significantly associated with the SRH scores of caregivers aged 65 years or older in the univariate linear regression model (Additional file 2: Appendix B; Table 2). In the multivariable analysis, the aforementioned variables were still significantly associated with SRH among caregivers aged 65 years or older. Caregivers aged 65 years or older who cared for care recipients that had used home nursing care rated their health higher than those who cared for care recipients that had not used the care service (β = 0.499, 95% CI = 0.146–0.852), adjusted for covariates. Moreover, unemployed caregivers aged 65 years or older showed a higher SRH score of 0.671 points compared with that of employed caregivers, after adjustment for independent and covariates. In addition, the SRH of care recipients had a significantly positive relationship with that of caregivers aged 65 years or older (β=0.222, 95% CI = 0.038–0.406), after adjusting for independent and covariates. However, caregivers aged 65 years or older who spent more than 80% of their family income on care showed a lower SRH score of 0.948 points than did those who spent 20% or less of their family income on care, after adjustment for independent and covariates.

## Discussion

The study provided empirical evidence on the effects of home-based LTC service use on caregiver health. We observed that home nursing care was positively associated with the SRH of caregivers aged 65 years or older. However, caregivers aged less than 65 years who cared for care recipients that had used home nursing care, rehabilitation and respite care showed poorer SRH than did those who cared for care recipients that had not used these services. The findings prove that the effects of home-based LTC service use on caregiver health may vary with caregiver age. In conclusion, age is a crucial factor for providing home-based LTC services.

Studies have reported that caregiver health was negatively related to age [12, 17, 18], and we also observed that after service use, the health of caregivers aged 65 years or older was significantly poorer than that of caregivers aged less than 65 years. The present study revealed a difference of 0.19 between the mean SRH scores of the caregivers aged 65 years or older and those aged less than 65 years. One previous study reported that the mortality rate increases by approximately 0.8% for every 1-point decrease in the SRH score [26]; therefore, the mortality rate of the caregivers aged 65 years or older was 0.15% more than that of the caregivers aged less than 65 years. Studies conducted in the United States and Japan have reported that formal care service use can reduce the caregiving burden on caregivers [13, 14]. In the United States, living in states with a higher or lower expenditure on home- and community-based services (HCBS) did not matter; caregiver stress among service users was lower than that among nonservice users [13]. In addition, the use of home care services had a negative relation to feelings of burden among family caregivers under the LTC insurance system in Japan [14]. Furthermore, caregiver health improves when caregiver burden decreases because burden is one of the major predictors of caregiver health [9, 12, 31]. Our study revealed that the use of home nursing care services can improve the health of elderly caregivers. The improved health of elderly caregivers may be attributed to a lower burden of caregiving after using formal care. In contrast, we observed that the health of younger caregivers was alleviated through use of home-based LTC services. The possible reason may be that younger caregivers were more likely to have job and cared for higher dependency level elders than older caregivers. Younger caregivers may have work and family conflicts because they must leave work and stay at home when formal caregivers provided home-based LTC services to care recipients. Additionally, the quantity of services subsidized in the TLTCP may be insufficient to reduce the burden of younger caregivers and stress resulted from caring for elders with a high dependency level, as reported by Kim and Yeom [32] for South Korea’s LTC insurance system. As a result, using home-based LTC services may not reduce the caring burden for younger caregivers and may cause an extra burden due to conflicts between work and family.

Our study revealed that daughters-in-law had more desirable health than did spouses, among caregivers aged less than 65 years. In Asia, daughters-in-law, rather than sons, commonly take the responsibility of caring for elderly people because of traditions regarding filial piety. When daughters-in-law cannot provide care, sons become the proxy caregivers. However, in some instances, spouses might not receive assistance from other family members because they live alone with the care recipients, due to a change in the family structure and filial piety [20–22]. A meta-analysis indicated that being a spouse was a risk factor for poor caregiver health [12]. Therefore, under formal LTC service, informal support is a supplementary method to improve caregiver health [4, 12, 33].

Care recipient health was positively associated with caregiver health regardless of caregiver age, as previously reported [11]. Care recipients who feel unhealthy may experience more physical, mental, and behavioural problems than those who feel healthy, and they require more care and living support from caregivers than those who feel healthy; thus, the poor health perception of care recipients may adversely affect caregiver health [6, 12, 15].

Our study revealed that a higher percentage of family income for care was associated with poorer health among elderly caregivers. The percentage of family income for care was an indicator of financial burden and was related to caregivers’ health [34]. Therefore, caregivers’ health could be elevated by reducing their financial burden. In addition, the study found that employed caregivers aged 65 or older were healthier than their unemployed counterparts. Prior studies confirmed the positive effects of employment on caregiver health [10, 35]. This may be that among elderly caregivers, working could ease the financial burden of caring through pay for work. Moreover, employed caregivers can possibly reduce the caring burden because they complete fewer caring hours and experience less social isolation than unemployed ones [36].

## Strengths and limitations

Our study has some strengths. The sample participants had experience using LTC services in the community and were not simply enrolled from clinical settings or LTC facilities. The results provided empirical evidence on the association between LTC services and the health of public caregivers. In addition, the present study compared the effects of four types of home-based LTC services and revealed the effectiveness of individual services. Moreover, the characteristics of caregivers and care recipients were simultaneously included in the analysis to control for possible confounders.

However, some limitations exist. First, the relationship between home-based LTC service use and caregiver health must be cautiously interpreted because of the cross-sectional study design. Future researchers can examine the robust effect of LTC service use on caregiver health through a longitudinal study design. Second, the study sample included care recipients from Taipei, Taiwan, and the results cannot be generalized to other cities in Taiwan. In addition, future research should include caregivers and care recipients from different countries and focus on how cross-cultural differences affect LTC service use and caregiver health. Even then, the results of the study still provided evidence on an association between LTC service use and caregiver health. Third, caregiver health was evaluated using a self-rated question. However, many studies have also used a single question to rate caregiver health [7, 9, 11, 37], and a study reported that SRH can predict mortality and medical care use [26]. Fourth, because physically ill or disability of caregivers could have an association with caregiver SRH [38], it is recommended that future studies should collect the comorbidity and disability of caregivers to alleviate the potential effect on health.

## Conclusion and suggestions

The findings of this cross-sectional study give a valuable indication that age-associated differences were observed for home-based LTC service use and caregiver health. It is notable that the use of home-based LTC services were negatively related to the SRH of caregivers aged less than 65 years. Relatively, among elderly caregivers, the percentage of family income for care was an important factor related to their health. Therefore, caregiver age needs to be taken into consideration to increase caregiver health and provide more effective and efficient home-based LTC services. Among younger caregivers, the quantity of home-based LTC services should be evaluated according to their need and caring burden. Nevertheless, for elderly caregivers, the need is to evaluate the financial burden of care and provide affordable LTC services. As a result, future studies can evaluate the long-term effects of home-based LTC services on caregiver health. In addition, the quantitative characteristics of home-based LTC services should be considered.

## Additional files

Additional file 1: Appendix A. Questionnaire. (DOC 58 kb)

Additional file 2: Appendix B. Univariate linear regression of effects of home-based long-term care service use on self-rated health among caregivers stratified by caregiver age. (DOCX 45 kb)

## Abbreviations

ADLs: Activities of daily living
HCBS: Home- and community-based services
IADLs: Instrumental activities of daily living
LTC: Long-term care
SF-36: Short form 36 health survey
SRH: Self-rated health
TLTCP: Ten-year long-term care project
