# Supplementary material for: Effects of home-based long-term care services on caregiver health according to age
Source: Health Qual Life Outcomes. 2017 Oct 23;15:208. doi: 10.1186/s12955-017-0786-6 (PMC5651602; doi:10.1186/s12955-017-0786-6)
Supplement: Supplementary file 1 — Questionnaire. (DOC 58 kb) [file 12955_2017_786_MOESM1_ESM.doc]

**Additional file 1: Appendix A: Questionnaire**

For caregiver

Please answer the following questions:

1. Age

1) <65

2) ≥65

2. Sex

1) Male

2) Female

3. Marital status

1) Unmarried

2) Married

4. Education level

1) Less than or equal to elementary school

2) Middle school

3) High school

4) College or above

5. Relationship with care recipient

1) Spouse

2) Son

3) Daughter

4) Daughter-in-law

5) Others

6. Quality of relationship with care recipient

1) Very bad

2) Bad

3) Good

4) Very good

7. Do you have job now?

1) Yes

2) No

8. Household monthly income (NTD)

1) <30,000

2) 30,000–69,999

3) ≥70,000

9. Family income spent on caring for the care recipient (%)

1) ≤20

2) 21–40

3) 41–60

4) 61–80

5) 81–100

10. How many years have you been caring for your care recipient? _____years

11. How would you rate your health generally since the care recipient started receiving LTC services?

1) Very poor

2) Poor

3) Fair

4) Good

5) Very good

For care recipients

Please answer the following questions:

1. Age

1) <65

2) 65–74

3) 75–84

4) ≥85

2. Sex

1) Male

2) Female

3. Marital status

1) Unmarried

2) Married

4. Education level

1) Illiterate

2) Literate/primary school

3) Junior high or above

5. Do you live alone?

1) Yes

2) No

6. How many family members lived with you?

1) 0

2) 1-3

3) ≥4

7. Quality of relationship with family

1) Very bad

2) Bad

3) Good

4) Very good

8. How would you rate your health generally since receiving LTC services?

1) Very poor

2) Poor

3) Fair

4) Good

5) Very good

9. Have you ever used home nursing care?

1) Yes

2) No

10. Have you ever used home rehabilitation?

1) Yes

2) No

11. Have you ever used home respite care?

1) Yes

2) No

12. Have you ever used home service?

1) Yes

2) No
