# Supplementary material for: Effects of home-based long-term care services on caregiver health according to age
Source: Health Qual Life Outcomes. 2017 Oct 23;15:208. doi: 10.1186/s12955-017-0786-6 (PMC5651602; doi:10.1186/s12955-017-0786-6)
Supplement: Supplementary file 2 — Univariate linear regression of effects of home-based long-term care service use on self-rated health among caregivers stratified by caregiver age. (DOCX 45 kb) [file 12955_2017_786_MOESM2_ESM.docx]

| Additional file 2: Appendix B Univariate linear regression of effects of home-based long-term care service use on self-rated health among caregivers stratified by caregiver age | | |
| --- | --- | --- |
| Variables | Caregivers aged less than 65  n=460 | Caregivers aged 65 or older  n=138 |
|  | β (95% CI) | β (95% CI) |
| Family caregivers |  |  |
| Sex |  |  |
| Male | Reference | Reference |
| Female | 0.029 (-0.160, 0.218) | -0.171 (-0.529, 0.186) |
| Marital status |  |  |
| Unmarried | Reference | Reference |
| Married | 0.005 (-0.216, 0.226) | 0.079 (-0.793, 0.952) |
| Education level |  |  |
| ≤ Elementary school | Reference | Reference |
| Middle school | 0.219 (-0.321, 0.759) | 0.095 (-0.446, 0.636) |
| High school | -0.018 (-0.462, 0.426) | -0.463 (-0.958, 0.032) |
| ≥College | 0.010 (-0.421, 0.441) | -0.296 (-0.786, 0.194) |
| Relationship with care recipient |  |  |
| Spouse | Reference | Reference |
| Son | 0.308 (0.006, 0.611)^*^ | 0.253 (-0.242, 0.748) |
| Daughter | 0.355 (0.063, 0.647)^*^ | -0.129 (-0.667, 0.409) |
| Daughter-in-law | 0.523 (0.184, 0.863)^**^ | 0.871 (-0.474, 2.216) |
| Others ^a^ | 0.211 (-0.237, 0.659) | 0.371 (-0.366, 1.108) |
| Quality of relationship with care recipient |  |  |
| Very bad | Reference | Reference |
| Bad | -0.383 (-1.375, 0.608) | 1.250 (-0.846, 3.346) |
| Good | 0.044 (-0.801, 0.888) | 0.667 (-1.225, 2.559) |
| Very good | 0.109 (-0.732, 0.950) | 0.671 (-1.216, 2.558) |
| Job |  |  |
| Yes | Reference | Reference |
| No | 0.279 (0.105, 0.453)^**^ | 0.646 (0.056, 1.236)^*^ |
| Household monthly income (NTD) ^b^ |  |  |
| <30,000 | Reference | Reference |
| 30,000-69,999 | 0.238 (-0.063, 0.539) | 0.108 (-0.451, 0.668) |
| ≥70,000 | 0.334 (0.033, 0.635)^*^ | 0.381 (-0.231, 0.993) |
| Family income spent on caring for the care recipient (%) |  |  |
| 20 or below | Reference | Reference |
| 21–40 | -0.227 (-0.458, 0.004) | 0.040 (-0.435, 0.515) |
| 41–60 | -0.200 (-0.446, 0.047) | -0.313 (-0.788, 0.162) |
| 61–80 | -0.304 (-0.660, 0.051) | -0.317 (-0.919, 0.284) |
| 81–100 | -0.647 (-1.031, 0.262)^**^ | -0.960 (-1.537, -0.383)^**^ |
| Caregiving period (years) | 0.012 (-0.004, 0.027) | -0.001 (-0.027, 0.026) |
| Care recipients |  |  |
| Age |  |  |
| 65 or less | Reference | Reference |
| 65–74 | -0.047 (-0.421, 0.327) | -0.106 (-0.981, 0.769) |
| 75–84 | 0.240 (-0.097, 0.577) | -0.152 (-0.976, 0.671) |
| 85 or older | 0.293 (-0.045, 0.630) | -0.159 (-0.987, 0.669) |
| Sex |  |  |
| Male | Reference | Reference |
| Female | 0.105 (-0.085, 0.295) | -0.151 (-0.507, 0.205) |
| Marital status |  |  |
| Unmarried | Reference | Reference |
| Married | 0.428 (-0.088, 0.944) | 0.190 (-0.612, 0.993) |
| Education level |  |  |
| Illiterate | Reference | Reference |
| Literate/Primary school | 0.018 (-0.235, 0.272) | 0.031 (-0.559, 0.622) |
| Junior high and above | -0.105 (-0.342, 0.131) | -0.534 (-1.072, 0.003) |
| Living alone |  |  |
| No | Reference | Reference |
| Yes | 0.323 (0.057, 0.589)^*^ | 0.466 (-0.194, 1.125) |
| Number of family members living with care recipient |  |  |
| 0 | Reference | Reference |
| 1–3 | -0.299 (-0.581, -0.017)^*^ | -0.599 (-1.250, 0.052) |
| 4 or more | -0.326 (-0.612, -0.040)^*^ | -0.075 (-0.785, 0.635) |
| Quality of relationship with family |  |  |
| Very bad | Reference | Reference |
| Bad | -1.071 (-2.493, 0.350) | 1.000 (-0.722, 2.722) |
| Good | -0.677 (-2.014, 0.660) | 0.855 (-0.503, 2.212) |
| Very good | -0.649 (-1.985, 0.687) | 0.534 (-0.822, 1.891) |
| Dependency level |  |  |
| Intact | Reference | Reference |
| Low | 0.100 (-0.388, 0.588) | 0.014 (-0.603, 0.632) |
| Moderate | -0.108 (-0.548, 0.331) | -0.192 (-0.755, 0.371) |
| High | 0.015 (-0.388, 0.588) | -0.058 (-0.827, 0.710) |
| SRH | 0.105 (0.007, 0.203)^*^ | 0.334 (0.152, 0.516)^***^ |
| Use of home-based services |  |  |
| Home nursing care | -0.253 (-0.440, -0.065)^**^ | 0.383 (0.022, 0.744)^*^ |
| Home rehabilitation | -0.302 (-0.520, -0.083)^**^ | 0.180 (-0.264, 0.624) |
| Home respite care | -0.301 (-0.575, -0.026)^*^ | -0.043 (-0.588, 0.503) |
| Home service | 0.217 (0.031, 0.404)^*^ | 0.130 (-0.232, 0.491) |

^a^ Others: son-in-law, grandchild, brother, sister, etc. ^b^ 30,000 NTD equals approximately 1,000 USD. CI=confidence interval; SRH=self-rated health. **p*<0.05; ***p*<0.01.
